# Hand hygiene compliance on two icus at hannover medical school: indication-specific analysis of compliance rates per bedside

**DOI:** 10.1186/2047-2994-4-S1-P157

**Published:** 2015-06-16

**Authors:** L Schwadtke, C Krauth, JT Stahmeyer, T von Lengerke, B Lutze, IF Chaberny

**Affiliations:** 1 Institute for Medical Microbiology and Hospital Epidemiology, Medical School Hannover, Hannover, Germany; 2 Institute for Epidemiology, Social Medicine and Health Systems Research, Medical School Hannover, Hannover, Germany; 3 Medical Psychology Unit, Medical School Hannover, Hannover, Germany; 4 Department of Diagnostics, Institute of Infection Control and Hospital Epidemiology, Leipzig Medical School, Leipzig, Germany

## Introduction

Health care-associated infections (HAIs) are a major problem on intensive care units (ICUs) [1]. Hand hygiene (HH) is considered to be the most important tool to prevent HAIs. The aim of this study was to generate indication-specific HH compliance focusing aseptic procedures (AP).

## Objectives

hand hygiene compliance, aseptic procedures.

## Methods

For a period of 2 weeks direct bedside observation (BO) was performed on a surgical ICU and a medical ICU in accordance to the WHO guideline “my 5 moments for hand hygiene” [2]: (1) “before contact with patients”, (2) before an AP”, (3) “after body fluid exposure”, (4) “after contact with patients”, and (5) “after contact with patients’ surroundings”. BO of HCW were performed from 7:00am to 7:00pm 3 days a week. AP were stratified into manipulation of ventilation devices (VD), intravascular catheters (IC), urinary catheters (UC), dressing (D), and other AP.

## Results

During the 144 hour observation period, a total of 1,896 opportunities for HH were observed for the two ICUs. The indication (2) was the most commonly observed indication (28.3%; n=537; see Fig.1). The overall HH compliance rate (CR) was 42.6%. The highest CR was evaluated for indication (4) (66.4%), whereas lowest CR was calculated for indication (2) (24.8%; see Fig. 2). Stratifying the AP into the different device manipulations described below revealed that “manipulation of IC” was the most frequently observed AP but only reached low CR (24.2%; n=293). The highest CR was evaluated for “manipulations of UC” (42.9%; n=14). In contrast lowest CR was observed with “manipulation of VD” (18.7%; n=134; see table 1).

## Conclusion

The overall CR per bedside was poor particularly with indication (2). Thus, future interventions to improve HCWs adherence to HH and therefore patients’ safety should focus on AP.

## Disclosure of interest

None declared.
